# The decreasing range between dry- and wet- season precipitation over land and its effect on vegetation primary productivity

**DOI:** 10.1371/journal.pone.0190304

**Published:** 2017-12-28

**Authors:** Guillermo Murray-Tortarolo, Víctor J. Jaramillo, Manuel Maass, Pierre Friedlingstein, Stephen Sitch

**Affiliations:** 1 Instituto de Investigaciones en Ecosistemas y Sustentabilidad, Universidad Nacional Autónoma de México, Morelia, México; 2 Cátedra CONACyT comisionado al Instituto de Investigaciones en Ecosistemas y Sustentabilidad, Universidad Nacional Autónoma de México, Morelia, México; 3 College of Engineering, Mathematics, and Physical Sciences, University of Exeter, Exeter, United Kingdom; 4 College of Life and Environmental Sciences, University of Exeter, Exeter, United Kingdom; University of Oregon, UNITED STATES

## Abstract

One consequence of climate change is the alteration of global water fluxes, both in amount and seasonality. As a result, the seasonal difference between dry- (p < 100 mm/month) and wet-season (p > 100 mm/month) precipitation (p) has increased over land during recent decades (1980–2005). However, our analysis expanding to a 60-year period (1950–2009) showed the opposite trend. This is, dry-season precipitation increased steadily, while wet-season precipitation remained constant, leading to reduced seasonality at a global scale. The decrease in seasonality was not due to a change in dry-season length, but in precipitation rate; thus, the dry season is on average becoming wetter without changes in length. Regionally, wet- and dry-season precipitations are of opposite sign, causing a decrease in the seasonal variation of the precipitation over 62% of the terrestrial ecosystems. Furthermore, we found a high correlation (r = 0.62) between the change in dry-season precipitation and the trend in modelled net primary productivity (NPP), which is explained based on different ecological mechanisms. This trend is not found with wet-season precipitation (r = 0.04), These results build on the argument that seasonal water availability has changed over the course of the last six decades and that the dry-season precipitation is a key driver of vegetation productivity at the global scale.

## Introduction

One consequence of anthropogenic climate change is the alteration of the hydrological cycle, causing greater water fluxes globally and changing current precipitation patterns [1]. As a result, moisture has increased over land in the wet regions/seasons and slightly decreased in the dry [2], although the actual change is complex, with high spatial and temporal variability [3,4].

Over the land, the range between dry- and wet-season precipitation has increased, particularly in the tropics [5,6], which has led to longer and more intense dry seasons in most arid ecosystems [2,7]. These results stem from the analysis of a relatively short-time period (e.g. 20 or 30 years of data), which may be insufficient to understand long-term changes in climate, as suggested in other studies [8]. Furthermore, recent research using annual data has proposed that the wetness/dryness trends in water availability and soil moisture do not seem to be related to the background climate [4], thus wet and dry regions can get wetter or drier depending on how atmospheric circulation patterns shift in space [9]. However, there has been no reconciliation of seasonal and annual trends over longer time periods (e.g. from mid-twentieth century).

In addition, recent research has shown an important link between changing seasonal and annual precipitation and trends in land carbon uptake [10]. In particular, attention has been focused on the role of dry ecosystems controlling the annual variability of land productivity [11,12] and on the impacts of change in the dry-season precipitation on regional NPP trends [7,13]. Apparently, dry regions, and particularly rainfall during the dry season, play an exceptionally important role in land carbon uptake.

In this study, we re-visited the changes in seasonal precipitation on land described by Chou et al. [5], but in two time periods: the original study period between 1979–2009 and an extended 60-year period between 1950–2009. We also complete the analysis with three observational products at a higher resolution (0.5º vs 2.5º in [5]). We explore whether the recorded seasonal changes documented for recent decades occur over a longer time period and explore the driving factors (changes in rate vs. changes in seasonal lengths). Our analysis also includes, the impact of regional changes in dry- and wet-season precipitation on evapotranspiration (ET) and runoff. Finally, we provide an ecological perspective to these trends by linking dry- and wet-season precipitation trends with modelled NPP from process-based simulations.

## Materials and methods

### Datasets

We used three observed precipitation datasets for the period 1950–2009, all at a 0.5º x 0.5º monthly resolution. These were: CRUV3.2 [14], GPCC [15] and PREC/L [16] (Table A in S1 File). These datasets are reconstructed based on gauge measurements of global observations and represent the best observations available, but contain limitations and uncertainties (S2 File). We used modelled ET, runoff and NPP from an ensemble of 9 DGVMs of the TRENDY-V1 compendium (Table B in S1 File) for the same period at a 1º x 1º monthly resolution [17]. All datasets were re-gridded to the same land-grid and are freely available online (Tables A and B in S1 File).

### Seasonal distribution of precipitation calculations

We computed the wet and dry seasons based on the number of months with less than 100 mm/month (dry-season precipitation) or with more than 100 mm/month (wet-season precipitation) of rain. This threshold was selected based on published studies dealing with multiple small-scale comparisons or with global observations [13,18–20]. For example, Zhang et al. [19] compared the partitioning of water fluxes (evapotranspiration and runoff) across more than 1000 small watersheds and found that on average, runoff occurs when monthly precipitation exceeds 80–120 mm/month; hence, this limit can be used to separate the hydrologically distinct seasons. Malhi et al. [20] and Doughty et al. [13] analysed the hydrological threshold across the Amazon and used a similar partitioning of ~100 mm/month. Finally, Chou et al. [5] used a running mean to calculate changes in global dry- and wet-season lengths, and found an average threshold between 4–6 mm/day. However, as the actual value to distinguish between seasons varies across the studies, we conducted a sensitivity test of the threshold with precipitation values of 50, 100, 150 and 200 mm (Table A in S3 File). Based on the results from local, regional and global scales, plus the sensitivity analyses, we justify the usage of a global precipitation threshold to separate the seasons; however, when analysing local conditions, the actual value should be adequate to better represent the climatology of the site.

The dry-season precipitation represented the sum of the total precipitation for all dry months, the dry-season length was the number of months with less than 100 mm/month of precipitation and the rate was simply the total dry-season precipitation divided by dry-season length (presented as mm/day). As in some regions multiple dry/wet seasons may occur within a year, we calculated the average number of dry seasons per grid and found that most regions on land have a single, well-constrained dry season (Fig B in S3 File). However, it is important to note that some regions may have more than one dry season within a year (e.g. Eastern USA, Northwestern Europe).

We plotted the mean annual dry-season, wet-season and total precipitation anomaly (each year minus the mean value for 1950–1960) for each year during 1950–2009, with each of the three precipitation products and for the ensemble mean. A simple linear regression and the uncertainty in the slopes were calculated, based on the standard deviation for all three products. The values for the slope (m) and the significance levels (p) are included in each plot.

The procedure was repeated with the mean annual dry-season length and the dry-season precipitation rate. The seasonal range was defined as the wet-season minus the dry-season precipitation trends, following the methodology of Chou et al. [5]. Mean values represent the average of all three products. This was done annually and spatially by pixel. In addition, we calculated the periodicity of wet or dry phases in the dry-season length and rate by doing time-series analysis decomposition. We employed a serial autocorrelation function (ACF) based on the global annual mean for the wet- and dry-season length.

The spatially-weighted correlations among all three variables were estimated, as well as the spatially-weighted percentage of negative and positive change in the seasonal range. Using the vegetation mask of Ramankutty and Foley [21], we calculated the area-weighted mean for the dry- and wet-season precipitations, and the seasonal range for each ecosystem type; these were ranked according to their mean annual precipitation. We included NPP gridded linear trends for climate only runs (TRENDY project [17] simulations Sim2-Sim1, where Sim2: driven by CO_2_+Climate and Sim1: CO_2_-only), and estimated a spatially-weighted correlation with the dry- and wet- season precipitation trends.

The trends in ET and runoff were determined from the Sim2 simulations. This was done for all 9 models, but results are presented only for the ensemble. We correlated the trend of all three variables (NPP, ET and runoff) with the trends in the dry- and wet-season precipitation, using a spatially-weighted correlation. Finally, we chose four areas with contrasting seasonal precipitation trends and compared them with the NPP trend in each case.

## Results and discussion

### Changes in the annual seasonal precipitation

**Fig 1:**
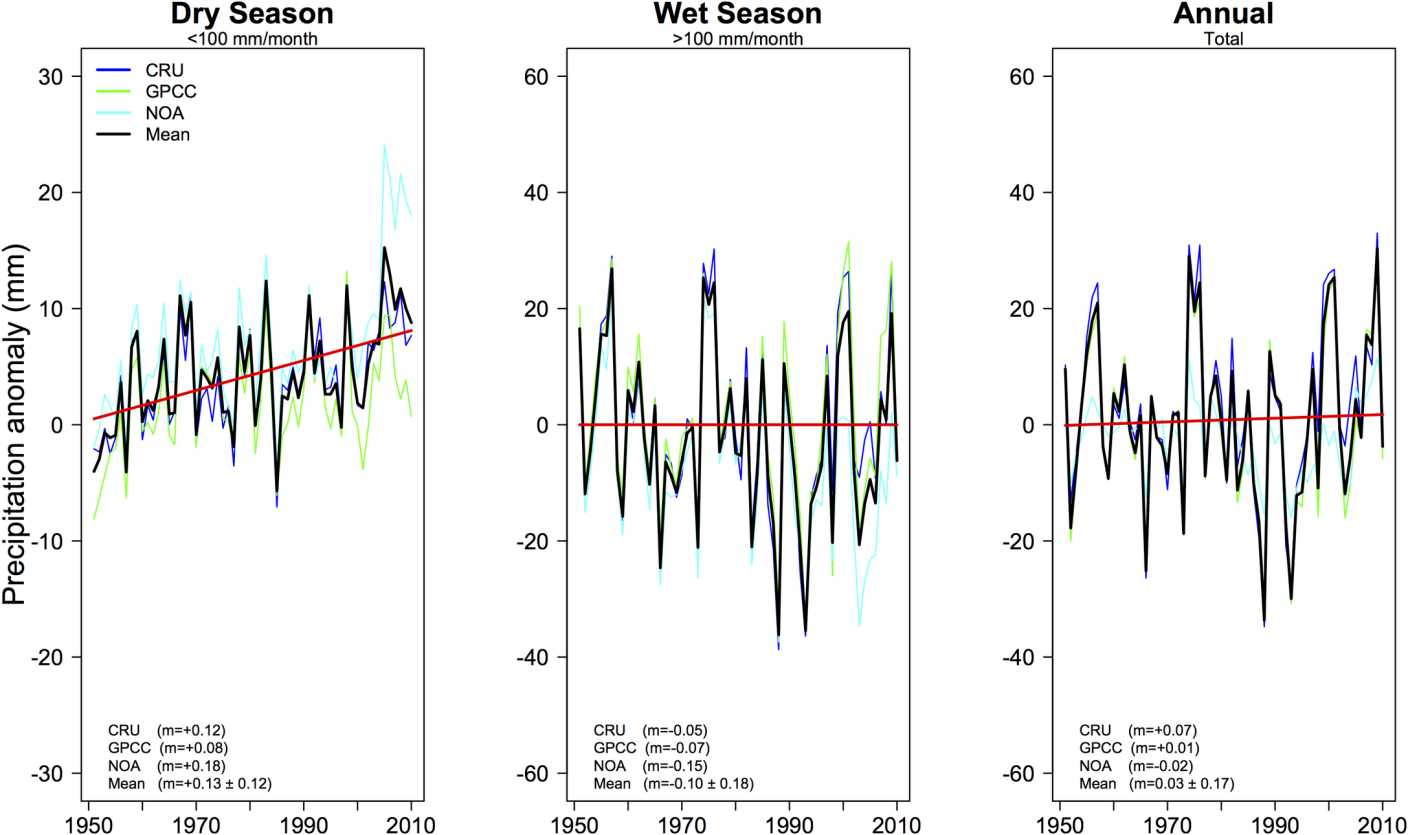
Mean seasonal and annual anomaly of global precipitation. Global annual dry-season precipitation (left), wet-season precipitation (middle) and annual precipitation (right) anomaly for 3 different precipitation products (CRU, GPCC, PRE/L) and the ensemble mean. Linear regression models for the ensemble mean are presented as a red line, and individual observation slopes are presented on the bottom left of each plot (m).

Our results showed that neither the annual nor the wet-season precipitation changed globally over the period 1950–2009, but there was a significant increase in dry-season precipitation (+0.13 ± 0.12 mm yr^-2^, p<0.001) (Fig 1). This resulted in a decrease in the global range between dry- and wet-season precipitation (-0.20 ± 0.9 mm yr^-2^) over land for the period 1950–2009, which was consistent across precipitation datasets (Table 1). These findings were opposite when the shorter time frame (1979–2009) was considered, with an increase in seasonal precipitation range (+0.20 ± 0.16 mm yr^-2^ in our study and +0.41 ± 0.16 mm yr^-2^, in Chou et al. [5]). Thus, it seems that the reported increase in the range between wet- and dry-season precipitation occurred only during the recent 25-year time period, but the trend acquired an opposite direction when computed for the longer 60-year window (Table 1). Apparently, the large change in wet-season precipitation is obscured in the longer time-frame and seems more likely a result of interannual or decadal variability, possibly due to a high number of La Niña years at the beginning of the 25-year time series (1988–1990) [22], the reduction of global precipitation caused by Mt. Pinatubo’s eruption in 1991, or it may represent the initial signal of the global effects of global warming on precipitation patterns. In contrast, the longer time-series shows a clear increase in dry-season precipitation, which is consistent across all observational datasets (Table 1). Previous studies over longer-time periods also reported a similar result and suggested that the increase in the dry-season precipitation could be linked to the loading of anthropogenic aerosols to the atmosphere [23], which have also been responsible for large changes in global atmospheric circulation [24].

**Table 1:** Wet, dry and seasonal range trends for a 30-yr period (same as Chou et al. [5]) and across half the 20^th^ century.

Period:	1979–2010 mm yr^-2^			1950–2009 mm yr^-2^		
Dataset	Wet	Dry	Range	Wet	Dry	Range
**CRU3.2**	0.44	0.13	+0.31	-0.03	0.12	-0.15
**GPCC**	0.41	0.05	+0.36	-0.03	0.08	-0.11
**NOA**	0.33	0.36	-0.03	-0.07	0.13	-0.20
**Mean**	0.38 ± 0.12	0.18 ± 0.20	**+0.20 ± 0.16**	-0.07 ± 0.4	0.13 ± 0.5	**-0.20 ± 0.9**
**Chou et al. [5]**	0.46 ± 0.17	0.05 ± 0.16	**+0.41 ± 0.16**			

### Mechanisms of change: seasonal length vs. seasonal precipitation rate

**Fig 2:**
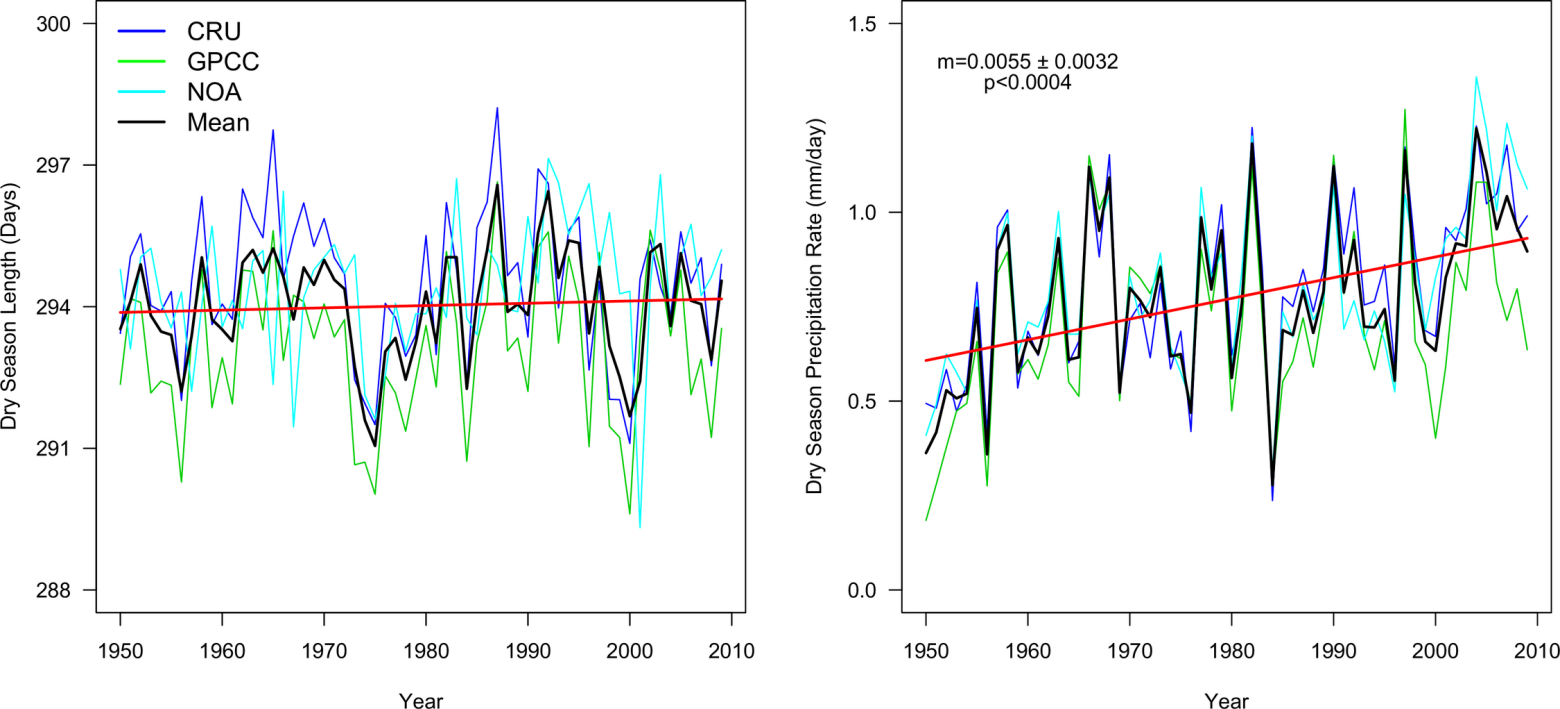
Global annual dry-season length (left) and precipitation rate (right). Legend and methodology follows Fig 1. The data employed is on a monthly resolution, but the figure is displayed in days for a better visualization.

There are two possible ways in which the dry-season precipitation may increase: a change in dry-season length, i.e. a longer dry season may accumulate more rain, and through an increase in precipitation rate, i.e. more rain accumulates over the same amount of time, or both. Our results showed that the mean global dry-season length remained similar from 1950–2009, lasting around 10 months on average (or 291–297 days based on the average of integer months multiplied by 30) (Fig 2A). However, there was strong decadal variability, with periods of increasing and decreasing length every 10–15 years (ACF = 13). In contrast, the dry-season precipitation rates increased steadily over time from 0.63 mm/day in 1950 to 0.88 mm/day in 2009, and displayed no consistent decadal variability (Fig 2B). The same mechanisms that modify rainfall seasonality globally operate regionally. We found small, mostly shortening changes in the dry-season length across a few key arid regions (e.g. South American and North American Plains), but strong regional changes in the dry-season precipitation rates (Fig A in S4 File). For example, there was no change in the dry-season length across most of the Amazon, but the rate increased steadily over the last 50 years.

Our results are consistent with recent findings showing a probable increase in the number of extreme daily precipitation events across dry regions [25,26], which could explain the increase in dry-season precipitation rates. Additionally, Sun et al. [23] found a decrease in global precipitation variability, with both the dry regions and dry seasons becoming wetter from 1940 to 2009. Thus, it seems that the length of the dry season has not changed globally during the last six decades, but the greater incidence of extreme weather events during the dry season has increased the daily precipitation rate, and thus, the dry seasons have become wetter on average. However, it is important to note that changes in forcing-mix could push the Earth-system in a different direction and thus, these hydrological trends may not be representative of what could occur in the future, particularly across extreme climate change scenarios. For example, aerosol-forcing was a key driver of global atmospheric trends during 1950–2009, but the signal is unlikely to remain into the future as positive greenhouse gas forcing dominates over negative aerosol forcing.

### Regional seasonal patterns

**Fig 3:**
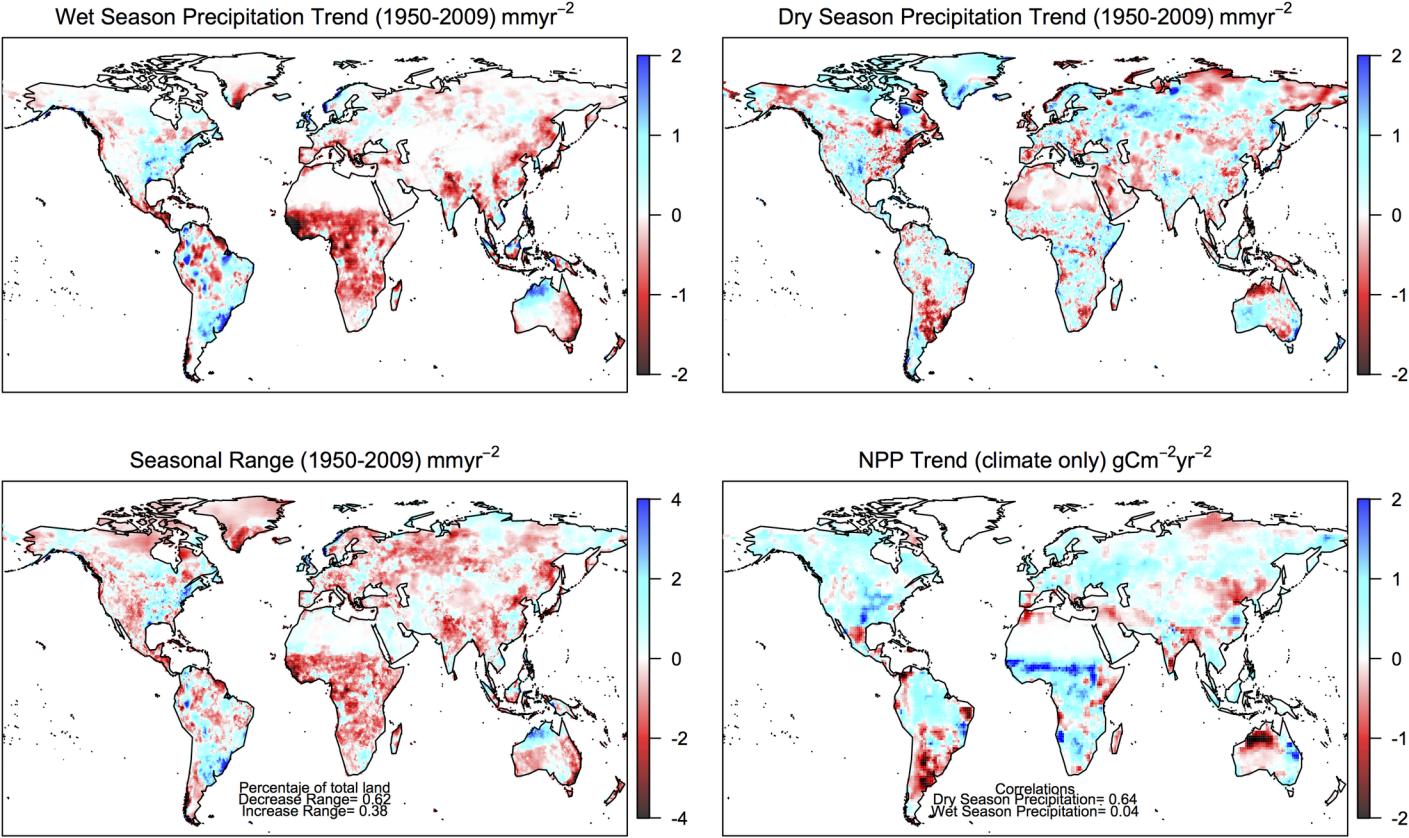
Global gridded seasonal precipitation and net primary productivity trends. Linear gridded trend for the wet- and dry-season precipitation, the seasonal range (wet minus dry) and vegetation net primary productivity (climate-only) for the period 1950–2009.

The decrease in the global seasonal range was also evident regionally. The gridded trends in wet- (Fig 3A) and dry-season (Fig 3B) precipitation were almost opposite to each other (r = -0.48): areas in which the wet-season precipitation increased, the dry-season precipitation decreased and vice versa. As a result, the seasonal range decreased in most of the land surface (excluding frozen regions) (62% of the total) (Fig 3C). The same pattern emerged in 10 out of 14 ecosystem types when the trend was aggregated by vegetation type, especially in the wetter ecosystems (Table B in S4 File). Overall, these results do not support the “dry gets dry” and “wet gets wet” hypothesis based solely on precipitation and are in agreement with current findings that drying and wetting patterns result from regional and local conditions [4].

### Impacts on water fluxes and vegetation productivity

Findings from 250 different watersheds with long-term data have shown that annual evapotranspiration correlates with precipitation on a 1:1 slope when precipitation is under ~1000 mm yr^-1^; beyond this threshold, the additional rain is lost via runoff [19]. Similar results are projected using the Budyko framework to partition water fluxes in terrestrial ecosystems [27,28]. Extrapolation of these results at a seasonal scale suggests that any alteration of the dry-season precipitation should lead to changes in evapotranspiration, thus affecting vegetation productivity [29]. In contrast, an increase or decrease in wet-season precipitation should translate only into changes in runoff.

We tested this prediction and found it robust at both the global scale for the period 1950–2009 (Fig A in S5 File) and over four regions with contrasting seasonal trends (Table B in S5 File). Changes in the wet-season precipitation were highly correlated with global runoff (r = 0.54), but only slightly with evapotranspiration (r = 0.16). In contrast, the dry-season precipitation trend was correlated to the evapotranspiration trend (r = 0.36), but not to runoff (r = 0.01). As a result, there was a strong correlation between the trend in modelled NPP and the trend in the dry-season precipitation (r = 0.64), mediated by its impact on evapotranspiration (Fig 3D). No such correlation was evident with the trend in the wet-season precipitation (r = 0.04) (Fig A and B in S6 File). This suggests that the vegetation responds to the excess or scarcity of dry-season rainfall. Productivity would be largely unaffected by changes in the wet-season precipitation, because a large proportion of this input does not circulate through the vegetation, but is lost via runoff. Also, vegetation growth during the wet season is usually limited by a factor other than moisture, like light availability or soil nutrients. For example, soil nutrient availability in seasonally-dry ecosystems increases mostly at the onset of rains, and other controls on available soil N, like soil C availability, may set in as the growing season progresses [30,31]; hence, productivity may be largely unaffected by changes in wet-season precipitation. These results, over a longer time period and at a higher resolution, are consistent with previous studies suggesting that changes in dry-season precipitation globally play a larger role controlling vegetation productivity than those occurring in wet-season precipitation [7]. Also, they support that vegetation greening of the planet may be partially driven by climate, in particular by interannual variability of precipitation over the tropics [32].

The relationship between dry-season precipitation and NPP may involve several plant physiological mechanisms that can vary across different ecosystems. For example, in the tropical wet forest, a reduction in water availability leads to hydraulic failure [33], which may lead to tree mortality and a decrease in NPP. Over water-limited semi-arid and arid ecosystems, the effects of changes in the hydrological regime are stronger [34,35]. For example, in the Amazon tropical forest leaf flushes and abscission are highly correlated with the length and intensity of the dry season [36]. A shorter dry season means longer foliated periods and rainfall during the dry season can cause re-foliation of many tree species [37]; as a consequence ecosystem NPP is increased [38]. Additionally, increased dry-season rainfall over arid regions may have two positive effects on vegetation: greater germination success of dominant annual species and longer growth periods [39]. On the other hand, a more intense dry season in arid grasslands dominated by annual species, does not only lead to a decrease in vegetation productivity, but also to a reordering of species abundance [40]. Therefore, across semi-arid and arid ecosystems, an increase in the dry-season precipitation represents a boost for dominant plant species productivity.

## Conclusions

Our study shows that the previously reported increase in the precipitation seasonal range across the land in recent decades (1989–2005) does not occur when a longer time-period is analysed (1950–2009). In fact, the trend is reversed, with the dry seasons getting wetter due to increased precipitation rates and with no change in the wet-season rainfall. Additionally, the modelled land NPP follows the trajectory of the dry-season precipitation across different time periods (1950–2009 and 1989–2005), which suggests a strong link between the dry-season rainfall and the land C cycle.

This work is relevant when considering future scenarios, particularly regarding the interaction between the hydrological and carbon cycles. Nevertheless, there are some limitations to this approach. First, the long-term trends in the past were driven by a different atmospheric forcing-mix than over recent decades and the future. For example, atmospheric loading was a key driver in the past, but forcing by greenhouse gases has become more dominant recently. This means that our results should be interpreted carefully if projected into the future. Secondly, our results are limited by the temporal resolution of the data. The use of monthly precipitation data implies that some of the analyses (e.g. dry season length) are too broad for local or regional studies. Thirdly, our analysis is based on the presence of a single well-constrained dry season. While this is true for most regions, some showed more than one annual dry-season. Although this does not affect the main conclusions globally, it may limit interpretations at a regional scale.

Finally, our study indicates that the link between precipitation seasonality and vegetation NPP is likely to operate at smaller temporal and spatial scales. Hence, site-level studies should employ higher resolution data, as they could provide further insights to the relationship between the hydrological and C cycle on land.

## Supporting information

S1 File Datasets employed in this work. (DOCX)

S2 File Data limitations and uncertainty. (DOCX)

S3 File Sensitivity analyses of dry/wet season metrics. (DOCX)

S4 File Gridded dry season precipitation rate and length trends. (DOCX)

S5 File Dry- and wet-season precipitation correlation to NPP in four contrasting regions. (DOCX)

S6 File Hydrological fluxes partitioning according to the level of precipitation and its effect on vegetation NPP. (DOCX)
