# Supplementary material for: The decreasing range between dry- and wet- season precipitation over land and its effect on vegetation primary productivity
Source: PLoS One. 2017 Dec 28;12(12):e0190304. doi: 10.1371/journal.pone.0190304 (PMC5746260; doi:10.1371/journal.pone.0190304)
Supplement: S1 File — (DOCX) [file pone.0190304.s001.docx]

**Supplementary 1: Datasets employed in this work**

Table S1.A List of observed precipitation datasets employed in this paper.

| **Dataset name** | **Abbreviation** | **Spatial Resolution** | **Source** |
| --- | --- | --- | --- |
| Climate Research Unit V3.2 | CR3.2 | 0.5ºx0.5º | Harris et al., 2014 |
| Global Precipitation Climatology Centre | GPCC | 0.5ºx0.5º | Schneider et al., 2014 |
| PRECipitation REConstruction over Land | PREC/L | 0.5ºx0.5º | Chen et al., 2012 |

Table S1.B. List of models used to calculate NPP, ET and runoff in this paper. A full description of the experiment and the runs, as well as the data can be found in Sitch et al. 2015.

| **Model Name** | **Abbreviation** | **Spatial resolution** | **Land Surface Model** | **Source** |
| --- | --- | --- | --- | --- |
| Community Land Model 4CN | CLM4CN | 0.5^o^×0.5^o^ | Yes | Oleson et al., 2010; Lawrence et al., 2011 |
| Hyland | HYL | 3.75^o^×2.5^o^ | No | Friend et al., 1997; Levy et al., 2004 |
| Lund-Potsdam-Jena | LPJ | 0.5^o^×0.5^o^ | No | Sitch et al., 2003 |
| LPJ-GUESS | LPJ-GUESS | 0.5^o^×0.5^o^ | No | Smith et al., 2001 |
| ORCHIDEE-CN | OCN | 3.75^o^×2.5^o^ | Yes | Zaehle& Friend, 2010; Zaehle et al., 2010 |
| ORCHIDEE | ORC | 0.5 ^o^×0.5^o^ | Yes | Krinner et al., 2005 |
| Sheffield-DGVM | SDGVM | 3.75^o^×2.5^o^ | No | Woodward et al., 1995 |
| TRIFFID | TRI | 3.75^o^×2.5^o^ | Yes | Cox, 2001 |
| VEGAS | VEGAS | 2.5 ^o^×2.5^o^ |  | Zeng et al., 2005 |
