# Supplementary material for: The decreasing range between dry- and wet- season precipitation over land and its effect on vegetation primary productivity
Source: PLoS One. 2017 Dec 28;12(12):e0190304. doi: 10.1371/journal.pone.0190304 (PMC5746260; doi:10.1371/journal.pone.0190304)
Supplement: S2 File — (DOCX) [file pone.0190304.s002.docx]

**Supplementary 2: Data limitations and uncertainty**

The main limitation of this study is that the CRU, GPCC and PREC/L datasets are not completely homogeneous. Many of the observations employed have been homogenized, but this was not done for all of them. Thus, the results from the trend analyses performed on these datasets should be treated cautiously, particularly across regions where observations are limited (e.g. high-latitudes, across most of Africa, and the deserts). Nonetheless, the homogenization and normalization processes, as well as the number of meteorological stations employed in each dataset are different, which makes them independent. Similarly, it is important to note that the data employed for NPP, runoff and ET come from model simulations and cannot be treated as observations. Therefore, results from these global analyses should be treated with caution when applied to a small region or to local conditions.
