# Supplementary material for: The decreasing range between dry- and wet- season precipitation over land and its effect on vegetation primary productivity
Source: PLoS One. 2017 Dec 28;12(12):e0190304. doi: 10.1371/journal.pone.0190304 (PMC5746260; doi:10.1371/journal.pone.0190304)
Supplement: S3 File — (DOCX) [file pone.0190304.s003.docx]

**Supplementary 3: Sensitivity analyses of dry/wet season metrics**

We conducted two tests to support our interpretation of the dry- and wet season trends. In the first one, the sensitivity of the global wet/dry season trends to the 100 mm threshold between seasons was evaluated. The second one estimates the average number of gridded dry seasons across the land during 1950-2009. Our results showed two important points: 1) when the threshold is changed by ± 50 mm (from a mean of 100 mm) the global trends in dry- and wet- seasons precipitation and the seasonal range are preserved, hence, it is likely that the seasonal range has decreased over the last 50 years, despite how the difference between seasons is defined. 2) When applied across the globe, our indices separate the seasons and most of the land showed 1 ± 0.5 dry seasons (average calculated excluding deserts and permafrost, where the number of dry seasons is 0).

Table S3.A Wet- and dry-season precipitation trends across terrestrial ecosystems for different thresholds. All calculations were done globally for the period 1950-2009 and represent the ensemble of the three observational products ± 1 standard deviation.

| **Threshold** | **Wet season trend** | **Dry season trend** | **Seasonal range trend** |
| --- | --- | --- | --- |
| **50 mm** | -0.04 ± 0.3 | 0.10 ± 0.8 | -0.14 ± 1.1 |
| **100 mm** | **-0.07 ± 0.4** | **0.13 ± 0.5** | **-0.20 ± 0.9** |
| **150 mm** | -0.02 ± 0.5 | 0.16 ± 0.9 | -0.18 ± 1.4 |
| **200 mm** | 0.06 ± 0.3 | 0.02 ± 0.8 | +0.04 ± 1.1 |

**
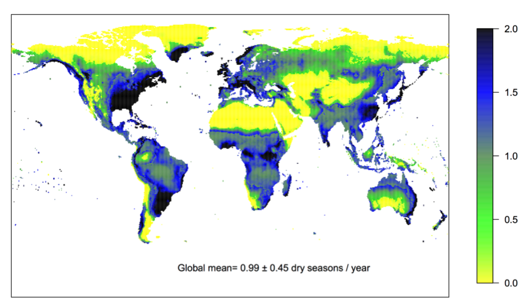
**

Figure S3.B Average number of dry seasons per year across the land. The mean number of dry seasons across the land is 1 dry season per year.
