# Supplementary material for: The decreasing range between dry- and wet- season precipitation over land and its effect on vegetation primary productivity
Source: PLoS One. 2017 Dec 28;12(12):e0190304. doi: 10.1371/journal.pone.0190304 (PMC5746260; doi:10.1371/journal.pone.0190304)
Supplement: S4 File — (DOCX) [file pone.0190304.s004.docx]

**Supplementary 4: Gridded dry season precipitation rate and length trends.**

Spatial trends for the dry-season length and the dry-season precipitation. Only small changes in the dry-season length were found, which contrasts with large changes in dry-season precipitation rates.


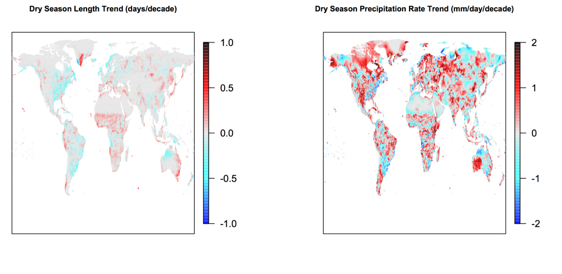


Figure S4.A Linear gridded trends for the dry-season length (left) and the dry-season precipitation rate (right).

We found a decrease in the global seasonal range by ecosystems. The trend in wet- and dry-season precipitation almost mirrored each other across all ecosystems and the dry-season precipitation was highly and positively correlated to the trend in NPP.

Table S4.B: Seasonal range trend by ecosystem and how it compares to the trend in NPP (climate-only).
