# Supplementary material for: The decreasing range between dry- and wet- season precipitation over land and its effect on vegetation primary productivity
Source: PLoS One. 2017 Dec 28;12(12):e0190304. doi: 10.1371/journal.pone.0190304 (PMC5746260; doi:10.1371/journal.pone.0190304)
Supplement: S5 File — (DOCX) [file pone.0190304.s005.docx]

**Supplementary 5: Dry- and wet-season precipitation correlation to NPP in four contrasting regions**

We tested the correlations between dry/wet-season precipitation and NPP over four regions with all possible combinations of precipitation trends (increase or reduction in either dry- or wet-season precipitation). As expected, regions where both dry- and wet-season precipitation increased, showed positive NPP trends. Where both trends decreased, NPP declined. Interestingly, in areas where the seasonal changes ran in opposite directions (e.g. an increase in dry-season precipitation and a decrease in wet-season precipitation) the change in NPP was driven by the dry season trend. This suggests that changes in vegetation productivity respond to the dry-season precipitation, regardless of the change in wet-season precipitation.

Figure S5.A: Four key regions of contrasting dry- and wet-season precipitation. For each one, the dry- and wet-season precipitation trends are represented by boxplots, the mean annual NPP as a black line and a linear regression model as a red line.

Table S5.B: Four key regions of contrasting dry and wet season precipitation trends and their link with NPP. The trend in dry-season precipitation is always positively correlated to NPP, while the trend in wet-season precipitation is only marginally correlated.

| Region | Longitude | Latitude | Dry Season Precipitation trend  (mm/yr^2^) | Wet Season Precipitation Trend  (mm/yr^2^) | NPP trend (gC/m^2^/yr^2^) |
| --- | --- | --- | --- | --- | --- |
| Tropical South America | 50-70 E | 10ºS-10ºN | 0.3 ± 0.1 | 0.4 ± 0.2 | 0.51 ± 0.32 |
| Mongolian Steppe | 110-120 E | 35-50 ºN | -2.6 ± 1.4 | -0.3 ± 0.1 | -0.37 ± 0.12 |
| Sahel | 20W-35E | 10-15ºN | 0.5 ± 0.2 | -2.4 ± 1.3 | 1.23 ± 0.45 |
| Argentinian Shrubland | 55-70 W | 30-40 ºS | -2.6 ± 0.8 | 3.7 ± 1.5 | -1.15 ± 0.78 |
