# Supplementary material for: The decreasing range between dry- and wet- season precipitation over land and its effect on vegetation primary productivity
Source: PLoS One. 2017 Dec 28;12(12):e0190304. doi: 10.1371/journal.pone.0190304 (PMC5746260; doi:10.1371/journal.pone.0190304)
Supplement: S6 File — (DOCX) [file pone.0190304.s006.docx]

**Supplementary 6: Hydrological fluxes partitioning according to the level of precipitation and its effect on vegetation NPP**

We found that changes in the wet-season precipitation were highly correlated with global runoff (r=0.54), but only slightly with evapotranspiration (r=0.16). In contrast, the dry-season precipitation trend was correlated to the evapotranspiration (AET) trend (r=0.36), but not to runoff (r=0.01). As a result, there was a strong correlation between the trend in NPP and the trend in the dry-season precipitation (r=0.64), mediated by its impact on evapotranspiration. No such correlation was evident with the trend in the wet-season precipitation (r=0.04).

Figure S6.A a) Partitioning of hydrological fluxes according to the level of precipitation as found in multiple watersheds and with the Budyuko framework. Precipitation under 100 mm month^-1^ is mostly transformed into AET; after this threshold it mostly turns into runoff. Linear gridded trend in the period 1950-2009 for b) wet-season precipitation, c) runoff, d) dry-season precipitation and e) runoff. Wet-season precipitation and runoff are highly correlated, as well as the dry-season precipitation and AET. As a consequence, there is a strong spatial correlation between the dry-season precipitation and the linear gridded trend in NPP (climate only).

Figure S6.B. Correlation of seasonal fluxes to components of the hydrological cycle and the link to global NPP trends. Derived from Figure S6
